# Localization of T cell clonotypes using the Visium spatial transcriptomics platform

**DOI:** 10.1016/j.xpro.2022.101391

**Published:** 2022-06-10

**Authors:** William H. Hudson, Lisa J. Sudmeier

**Affiliations:** 1 Emory Vaccine Center, Atlanta, GA, USA; 2 Department of Microbiology and Immunology, Emory University School of Medicine, Atlanta, GA 30322, USA; 3 Department of Radiation Oncology, Emory University School of Medicine, Atlanta, GA 30322, USA; 4 Winship Cancer Institute, Emory University School of Medicine, Atlanta, GA 30322, USA

**Keywords:** Sequence analysis, Immunology, Molecular Biology

## Abstract

We present a protocol to localize T cell receptor clones using the Visium spatial transcriptomics platform. This approach permits simultaneous localization of both gene expression and T cell clonotypes in situ within tissue sections. T cell receptor sequences identified by this protocol are readily recapitulated by single-cell sequencing. This technique enables detailed studies of the spatial organization of the human T cell repertoire, such as the localization of infiltrating T cell clones within the tumor microenvironment.

For complete details on the use and execution of this protocol, please refer to Sudmeier et al. (2022).

## Highlights

• Modification of the Visium platform allows parallel spatial mRNA and TCR sequencing
• Localize T cell receptor (TCR) sequences *in situ* within tissue sections
• Spatial and single-cell sequencing yield similar TCR sequences

## Abstract (editor highlights)

Publisher's note: Undertaking any experimental protocol requires adherence to local institutional guidelines for laboratory safety and ethics.

## Graphical abstract

**Figure:**
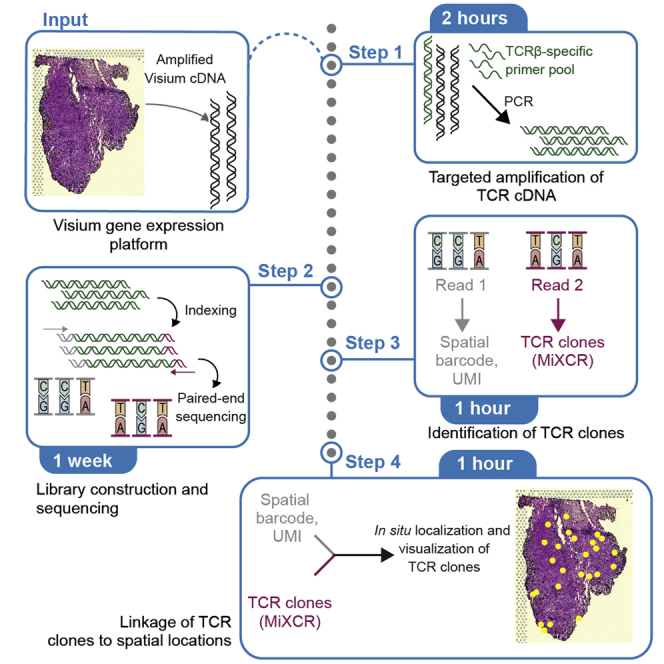

## Before you begin

This protocol describes a method for determining the localization of human T cell receptor (TCR) sequences using 10x Genomics’ Visium Spatial Gene Expression platform. This method uses targeted amplification of TCRs from cDNA generated by the Visium Spatial Gene Expression assay. Thus, before beginning, appropriate human tissue should be frozen and embedded, cryosectioned onto a Visium Gene Expression Slide, stained with hematoxylin and eosin (H&E) or immunofluorescence antibodies, and imaged with an appropriate microscope. cDNA should then be generated from the Visium Gene Expression Slide according to the manufacturer’s protocols. Downstream analysis of TCR sequences with the sample software code presented here requires generation and sequencing of the matched Visium gene expression cDNA and analysis with 10x Genomics Space Ranger software.

Here, we embedded freshly-resected human brain metastasis samples in OCT and froze them according to the manufacturer’s protocols. 10 μm tissue sections were placed onto the Visium Gene Expression Slide, stained with H&E according to the manufacturer’s protocols and imaged with a Cytek Lionheart microscope at 10× magnification in color brightfield mode. The manufacturer’s detailed, published protocols were followed to generate cDNA.

**Figure 1:**
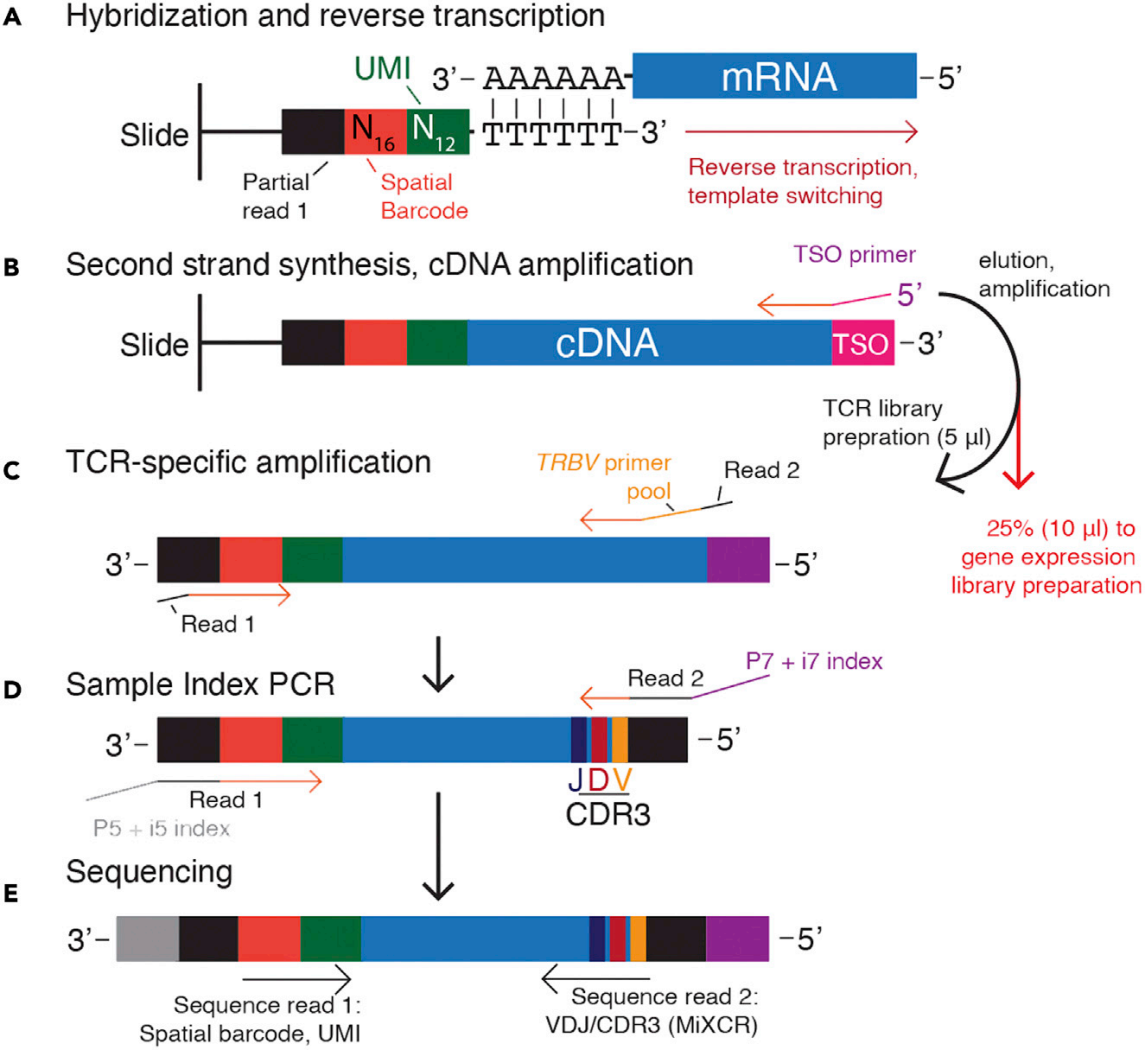
A method for obtaining TCR sequences from the Visium spatial transcriptomics assay See text (“Before you begin”) for details.

The Visium spatial transcriptomics platform uses slide-bound, single-stranded DNA probes to capture polyadenylated mRNA. Each probe contains a partial read 1 sequence at the 5′ end, a 16-nucleotide spatial barcode, a 12-nucleotide unique molecular identifier (UMI), and a 3′ poly(dT) tail (Figure 1A). The read 1 primer sequence is initially used for cDNA amplification; the spatial barcode links the probe to a particular spatial location; and the UMI identifies unique cDNA molecules generated during first-strand synthesis. After imaging and tissue permeabilization, polyadenylated RNA molecules anneal to the poly(dT) tail, allowing subsequent reverse transcription to extend the probe to contain the cDNA sequence and an added template switching oligo (TSO) (Figure 1A). The second strand is generated with a primer complementary to the TSO (Figure 1B); addition of potassium hydroxide elutes this full-length second strand from the slide. The eluted second strand is amplified to generate a full-length cDNA library with the spatial barcode and UMI to the 3′ end of the poly(A) tail (Figure 1B).

This cDNA from a Visium Gene Expression Slide is generated in a 40 μL volume (step 3 of (10x Genomics, 2022)); 10 μL is used for downstream generation of the gene expression library (10x Genomics, 2022). Thus, 30 μL of cDNA is available for targeted amplification studies such as the TCR method presented here. To generate cDNA molecules containing both the CDR3 region of the TCRβ as well as the UMI and spatial barcodes containing the location information, TCRβ amplification must occur with primers at the 5′ end of the TCR (Figures 1C and 1D). Since the TCRβ constant region is 3′ to the CDR3, a pool of variable (*TRBV*) gene primers is required to generate cDNA molecules containing both the CDR3 and spatial information (Figure 1C). To generate human libraries, we used primers containing a partial Ilumina Read 2 sequence joined at the 5′ end to each of a set of 45 human *TRBV* primers described previously (Table S1) (Robins et al., 2009). PCR is performed with this pool of modified *TRBV* primers and a Read 1 primer using amplified cDNA from the Visium assay as template to generate TCR-enriched libraries (Figure 1C). While the standard Visium gene expression protocol calls for fragmentation of the cDNA library, TCR-enriched libraries generated here cannot be fragmented without losing linkage between TCR and spatial barcode sequence. Thus, the TCR-enriched cDNA library generated here was cleaned with bead purification and directly subjected to sample index PCR (Figure 1D) for multiplexed paired-end sequencing (Figure 1E).

After sequencing, read 1 will contain the N_16_ spatial barcode and N_12_ UMI sequences, and read 2 will contain the TCR CDR3 region. MiXCR (Bolotin et al., 2015) is used to identify TCR clones and the sequences from read 2 that support each clone. The paired read 1 for each TCR-containing read 2 is used to localize the TCR read with the spatial barcode and correct for PCR duplication with the UMI. An outline of this process is shown in Figure 1.

While we describe a protocol to detect human TCRβ sequences, this method should be applicable to TCRα sequences by using primer pools specific for human *TRAV* genes (Robins et al., 2009). Primer pools specific to variable T cell receptor genes of other species should allow this method to be applied to tissue sections from other species such as mouse (Dash et al., 2011; Saligrama et al., 2019). Additionally, the method may also be generalized to obtain B cell receptor sequences: Igκ- and Igλ-specific primer pools (Tiller et al., 2008; von Boehmer et al., 2016) should generate libraries with insert sizes comparable to the TCR libraries described here, whereas sequencing of heavy chains may require long-read sequencing.

Prior to performing this protocol, 5 μL of amplified cDNA from the Visium Gene Expression Slide is required.

### Generation of Visium Spatial Gene Expression cDNA

**Timing: 1–2 days**

1. 5 μL of Visium gene expression cDNA is required for this protocol. This should be generated according to the manufacturer’s instructions:
  a. Tissue should be frozen, embedded, and cryosectioned on a Visium Gene Expression Slide.
  b. Tissue should be methanol fixed, stained with immunofluorescence antibodies or H&E stain and imaged with an appropriate microscope. Results shown here are from H&E staining imaged with a Cytek Lionheart in color brightfield mode at 10× magnification.
  c. Following imaging, cDNA should be generated according to the manufacturer’s protocol.

### Generation of TCRβ primer pool

**Timing: 10 min (hands-on)**

2. Order 45 human *TRBV* specific primers with partial read 2 sequences at the 5′ end.
  a. Sequences are given in Table S1. We recommend ordering these primers from Integrated DNA Technologies in a 96-well plate format with the complete yield resuspended to 100 μM.
3. Combine these 45 *TRBV*-specific primers in equimolar amounts.
  a. If ordered as in step 2a above, equal volumes of each primer can be added to a single tube.
4. Dilute *TRBV* primer pool to a total concentration of 10 μM (0.22 μM of each primer) using nuclease free water.
  a. If prepared as in steps 2 and 3 above, add nuclease-free water at a 9:1 ratio to the pooled primers (e.g., 90 μL water to 10 μL primer pool).
  b. This primer pool is stable at 4°C for up to 2 weeks.

### Institutional permissions

The permission of a relevant Institutional Review Board or similar body should be obtained before conducting any studies on human samples. These experiments were performed with the approval of the Emory University Institutional Review Board under protocols IRB00045732, IRB00095411, and STUDY00001995. Experiments on animal tissue should be approved by an Institutional Animal Care and Use Committee or similar body.

## Key resources table

**Table:** 

REAGENT or RESOURCE	SOURCE	IDENTIFIER
**Biological samples**		

Amplified cDNA from step 3.4 of the Visium Spatial Gene Expression Reagent Kits User Guide	User	n/a

**Chemicals, peptides, and recombinant proteins**		

Nuclease-free water	Thermo Fisher Scientific/Ambion	Cat# AM9932
Library Construction Kit	10x Genomics	Cat# PN-1000190
SPRIselect beads	Beckman Coulter	Cat# B23317
Buffer EB	QIAGEN	Cat# 19086

**Critical commercial assays**		

Bioanalyzer with high sensitivity DNA kit and reagents	Agilent	Cat# 5067-4626
Qubit instrument with dsDNA HS Assay kit (optional)	Thermo Fisher Scientific	Cat# Q32854

**Oligonucleotides**		

*TCRB* + read 1 primers and read 2 primer (see Table S1 for sequences)	Integrated DNA Technologies	n/a
Dual Index Kit TT Set A sample index primers	10x Genomics	Cat# PN-1000215

**Software and algorithms**		

MiXCR (Bolotin et al., 2015)	GitHub or Homebrew	https://github.com/milaboratory/mixcr or https://mixcr.readthedocs.io/en/master/install.html
Example spatial TCR sequencing code	This paper and Sudmeier et al., *Cell Reports Medicine* 2022	https://github.com/whhudson/spatialTCR (https://doi.org/10.5281/zenodo.6368907)

**Deposited data**		

scRNA-seq data	Sudmeier et al., *Cell Reports Medicine* 2022	GEO: GSE179373
Spatial transcriptomics data	Sudmeier et al., *Cell Reports Medicine* 2022	GEO: GSE179572
Spatial TCR-sequencing reads	Sudmeier et al., *Cell Reports Medicine* 2022	BioProject: PRJNA742564

**Other**		

Magnetic Separator for PCR tubes	10x Genomics	Cat# 120250
Thermocycler (e.g., Nexus GX2)	Eppendorf	Cat# 6336000023
Widefield color brightfield microscope (e.g., Lionheart FX)	BioTek	n/a
Sequencer (e.g., Illumina MiSeq)	Illumina	n/a

## Step-by-step method details

### Amplification of TCR sequences from Visium cDNA

**Timing: 2 h**

This protocol begins with Visium cDNA, which contains amplified cDNA of all genes expressed in the target tissue section. In these steps, we use *TRBV*-specific primers to amplify TCRβ cDNAs from the Visium cDNA pool (Figure 1C).

1. Prepare TCR cDNA amplification mix by adding the following components in a PCR tube:

**Table:** 

Reagent	Amount
Amplified Visium cDNA	5 μL
Amplification mix (from 10x Genomics Library Construction Kit)	7.14 μL
*TRBV* primer pool (from “Generation of TCRβ primer pool” above; final 200 nM)	0.29 μL
10 μM Partial read 1 primer (final 200 nM)	0.29 μL
Nuclease-free water	1.57 μL

2. Mix gently by pipetting up and down; centrifuge briefly with a mini centrifuge.
3. Amplify TCR cDNA in a thermocycler with the following cycling conditions:

**Table:** 

Steps	Temperature	Time	Cycles
Initial Denaturation	98°C	3 min	1
Denaturation	98°C	15 s	35 cycles
Annealing	59°C	20 s	
Extension	72°C	1 min	
Final extension	72°C	1 min	1
Hold	4°C	forever	

### Bead cleanup

**Timing: 30 min**

This step uses size selection to purify the TCR-enriched PCR product, removing primers and other reaction components that may interfere with library preparation.

4. After amplification, bring PCR reaction volume up to 50 μL by adding 35.7 μL of nuclease-free water.
5. Ensure the SPRIselect beads are resuspended and mixed well by shaking.
6. Add 0.6× (30 μL) of SPRIselect beads to the PCR product from step 4.
7. Mix well by pipetting.
8. Incubate 5 min at room temperature.
9. Place on separation magnet until solution clears.
10. Pipette to remove and discard supernatant.
11. Add 200 μL of 80% ethanol to the pellet. Do not mix or remove tube from magnet. Wait 30 s.
12. Pipette to remove and discard supernatant.
13. Repeat steps 11 and 12 once, resulting in two 80% ethanol wash steps.
14. Ensure that all 80% ethanol is removed. Air dry for two minutes.
15. Remove tube from magnet.
16. Add 40.5 μL Buffer EB to tube. Resuspend beads gently by pipet mixing.
17. Incubate 2 min at room temperature.
18. Place on separation magnet until solution clears.
19. Transfer 40 μL to a new PCR tube. This is the amplified TCR cDNA.

**Pause point:** Purified, amplified TCR cDNA may be stored at 4°C for 24 h or frozen at −20°C for longer storage (at least 1 month).

***Optional:*** Purified, amplified TCR cDNA can be quantified using a Qubit High Sensitivity Assay kit according to manufacturer’s instructions. Our yields ranged from 50–150 ng of total product. Alternatively, product may be analyzed using a Bioanalyzer or similar high-sensitivity electrophoresis platform.

### Sample indexing PCR

**Timing: 2 h**

This step creates sequencer-ready, dual-indexed libraries by adding Illumina P5 and P7 sequences, i5 and i7 indices, and completing the TruSeq Read1 and Read2 sequences on the amplified TCR cDNA (Figure 1D).

20. Prepare sample indexing PCR reaction for library generation and multiplexed sequencing in a PCR tube as outlined below:

**Table:** 

Reagent	Amount
Amplified TCR cDNA from step 19	15 μL
TT set A primers (from one individual well)	10 μL
Amplification mix (from 10x Genomics Library Construction kit)	25 μL

  a. Record the well used from the TT set A primers plate; this will allow for demultiplexing of pooled TCR sequencing libraries.
21. Amplify the mixture with the following conditions:

**Table:** 

Steps	Temperature	Time	Cycles
Initial Denaturation	98°C	45 s	1
Denaturation	98°C	20 s	35 cycles
Annealing	67°C	30 s	
Extension	72°C	1 min	
Final extension	72°C	1 min	1
Hold	4°C	forever

22. Perform cleanup of PCR product as described in “bead cleanup” section above (steps 4–19). The final product is the spatial TCR library.

**Figure 2:**
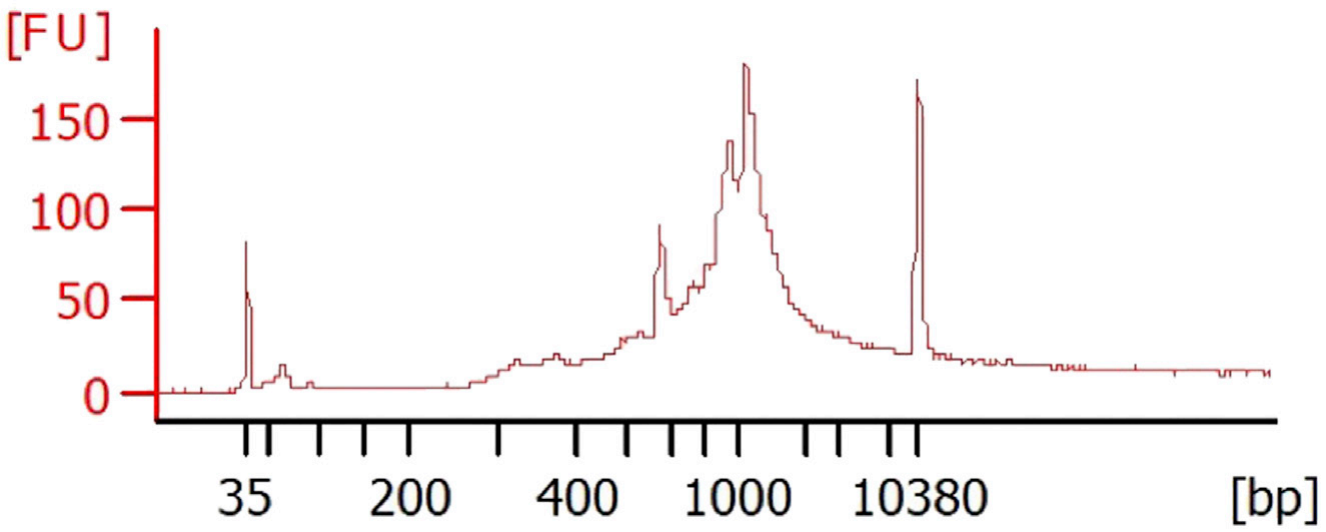
Bioanalyzer trace of pooled spatial TCR libraries

***Note:*** TCRβ cDNA amplified via this method will average around 1 kb in length. We typically achieved total library yields of 1–2 μg. See Figure 2 for an example Bioanalzyer trace. TCRβ cDNA was diluted 1:10 before Bioanalzyer analysis.

### Library quantification and sequencing

**Timing: About 1 week**

This step will quantify and sequence the spatial TCR library. As long as different dual-indexing primers are used for each library in step 20, multiple libraries can be sequenced simultaneously.

23. Run 1 μL of a 1:10 dilution of the spatial TCR library on a Bioanalyzer or similar high-sensitivity electrophoresis platform.
24. Sequence libraries according to the manufacturer’s instructions. We performed sequencing on an Illumina MiSeq using a MiSeq Reagent Kit v3 at PE300 with 10% PhiX. Libraries were loaded at 20 pM concentration.

***Note:*** Spatial TCR libraries are expected to contain low sequence diversity, thus the use of 10% PhiX. Paired end, dual indexed sequencing must be performed to generate TCR sequences (Read 2) and spatial barcode and UMI information (Read 1). Read 1 must be sequenced for at least 28 cycles to obtain full spatial barcode and UMI sequences; we recommend a read length of at least 100 nucleotides for read 2.

### Identification of TCR clones

**Timing: 1 h**

Paired-end sequencing from step 24 will result in the generation of two FASTQ files. Read 1 contains the spatial barcode and UMI sequences, and read 2 contains the TCR VDJ region sequences (Figure 1E). Here, we use MiXCR (Bolotin et al., 2015) to call antigen receptor sequences from read 2. We will also export reads supporting each TCR in order to identify paired reads that contain spatial barcode and UMI information. Example code, sample sequencing files, and expected MiXCR output is provided at https://github.com/whhudson/spatialTCR.

Files needed:

Read 2 FASTQ file(s) from ”library quantification and sequencing“ section, above.

**Software needed**: MiXCR (version 3.0.13 is the latest release as of writing). See key resources table for URLs for download.

25. Use the mixcr analyze pipeline to call antigen receptor sequences from the read 2 FASTQ file.
  a. The following code is provided as an example. path/to/read2.fastq is the file path to the read 2 FASTQ file generated from step 24.
    mixcr analyze shotgun -s hsa --starting-material rna --align "-OsaveOriginalReads=true" --assemble "--write-alignments" path/to/read2.fastq.gz mixcr_output/clones
  b. If performed as written above, output will be stored in the mixcr_output/ folder with file prefix “clones”.
    ***Note:*** MiXCR analyze parameters used are the following: the “-s” option designates the species of organism; “hsa” indicates *Homo sapiens*. The “--starting material” option designates the type of nucleic acid from which libraries were made, RNA in this protocol. ‘--align “-OsaveOriginalReads=true”’ forces MiXCR to save the original sequencing reads for use in later steps. ‘--assemble “--write-alignments”’ saves the alignments and clone mapping to a .clns file that is used in step 26.

26. Export individual reads supporting each clone with the mixcr exportReadsForClones command.
  a. The following code is provided as an example.
    mixcr exportReadsForClones -s mixcr_output/clones.clns mixcr_output/reads/reads.fastq.gz
  b. If performed as written above, this will result in fastq.gz files with reads supporting each clone being written into the mixcr_output/reads/ folder as reads_clnN.fastq.gz, where N is the clone number identifier.
    ***Note:*** The “-s” parameter in MiXCR exportReadsForClones forces MiXCR to make separate read files for each clone. The mixcr_output/clones.clns file is generated in step 25.
    ***Note:*** At this point, TCR clonotypes have been identified from individual sequencing reads. Information about each clone is provided in the mixcr_output/clones.clonotypes.ALL.txt file if the naming convention above has been followed. Additionally, the sequencing read 2 for each clone has been written into the mixcr_output/reads folder.

### Linkage of TCR clones to spatial locations

**Software needed**: Python 2 (for sample code).

Now, the UMI and spatial barcode should be extracted from each paired read 1 of TCRs identified in the previous step. This allows mapping of each TCR read to a UMI and spatial location. Duplicate reads with identical UMIs should also be discarded, such that each UMI and spatial barcode combination is only used once in downstream analysis. We provide the example Python 2 script combine_counts.py at https://github.com/whhudson/spatialTCR for performing these tasks.

27. In the provided combine_counts.py script, specify the directory containing the reads written by MiXCR (path/to/mixcr_output/reads if using file naming as above), the tissue positions list from 10x Genomics Space Ranger output, and the path to the read 1 FASTQ.gz file from the sequencing step above.
28. Running the script will output a tab-delimited text file with each spot/spatial barcode as a row and each clone as a column. The number in each cell indicates the number of UMIs measured in each spatial barcode (row) for a particular clone (column). This tab-delimited text file can then be used for further analysis in R, such as to add metadata to a Seurat spatial object.

## Expected outcomes

We performed this protocol and analysis on six fresh-frozen tissue sections of resected human brain metastases (Table 1). These samples were drawn from four primary tumor types with varying levels of T cell infiltration (Sudmeier et al., 2022).

**Table 1:** Results from spatial TCR sequencing on six human brain metastasis samples

	Sample 15	Sample 16	Sample 19	Sample 24	Sample 26	Sample 27
# reads	509,157	2,057,866	348,486	754,089	1,130,748	2,993,934
#TCR UMIs	590	1973	1	135	21	411
# clones	24	113	1	44	7	84
Diversity (Shannon)	1.82	2.27	0	3.19	1.32	3.89
TCR sequencing saturation	98.4%	98.1%	>99.9%	98.7%	>99.9%	99.9%
TCR UMIs/spot	0.28	1.15	0.001	0.10	0.005	0.19
*CD3E* counts (Visium; untargeted)	362	328	16	81	18	98
CD8^+^ cells/g tumor (×10^5^)	3.76	23.07	0.87	0.96	1.78	30.38
Primary tumor type	Lung	Melanoma	Lung	Renal clear cell	Breast	Lung

**Figure 3:**
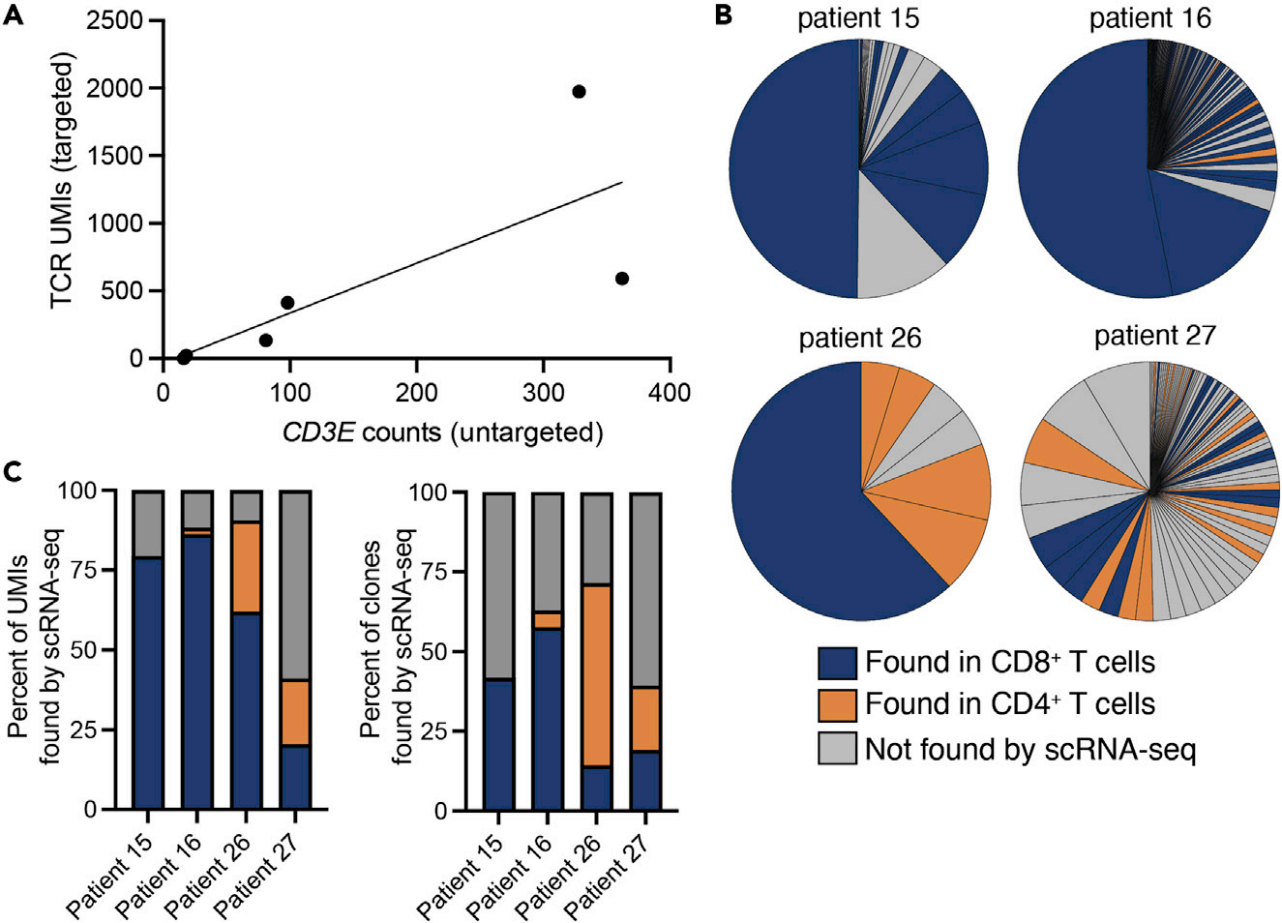
TCRβ sequences obtained from Visium are recapitulated by single-cell RNA-sequencing (A) Correlation of TCR sequences obtained from the assay with expression of *CD3E* (a pan-T cell marker) by Visium gene expression analysis. (B) For four patients, single cell RNA-sequencing (scRNA-seq) on T cells was performed to validate TCR sequences obtained by the spatial assay (Sudmeier et al., 2022). Pie charts show frequency of clones obtained from the spatial assay and are colored based on whether the clones were independently found through scRNA-seq in CD4^+^ or CD8^+^ T cells. (C) Summary of clone recovery statistics. Note: CD4^+^ T cells and PD-1^-^ CD8^+^ T cells were not sequenced from patient 15 due to lack of cryopreserved cells.

The number of TCR UMIs identified with this method ranged from 1 to 1,973 per sample, and TCR recovery was loosely correlated both with *CD3E* transcript counts by the standard Visium gene expression assay and CD8^+^ T cell infiltration by flow cytometry (Figure 3A and Table 1). The number of unique clones identified ranged from 1 to 113 and diversity (Shannon index) from 0 to 3.89 (Table 1 and Figure 3B).

To determine whether the TCRs identified by our technique were recapitulated by other methods, we performed single cell RNA-sequencing (scRNA-seq) on T cells from four matched patients (Sudmeier et al., 2022). CD8^+^ T cells were sequenced from all patients, and CD4^+^ T cells were sequenced from three of the four. On average, TCRs from 74.8% of UMIs identified by the spatial method described here were also identified in matched scRNA-seq samples with the cellranger VDJ pipeline (Figure 3C). TCR recovery between spatial and scRNA-seq TCR was lower for patient 27; this is likely due to higher spatial TCR diversity in this sample (Table 1) and a lower number of cells sequenced by scRNA-seq. Thus, if performing scRNA-seq on T cells from matched samples, the number of cells sequenced should be increased for particularly diverse samples.

**Figure 4:**
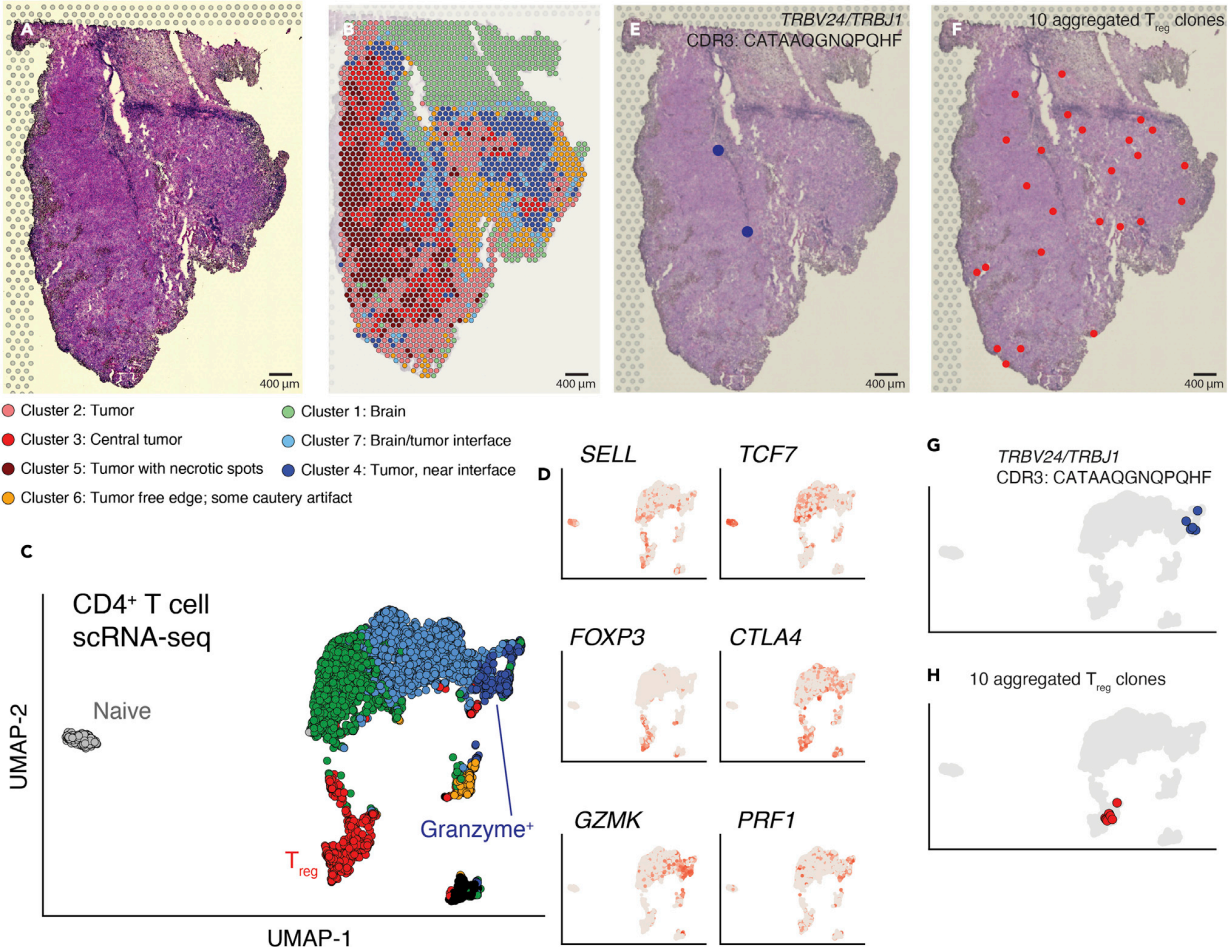
Example spatial localization of CD4^+^ T cell clones (A) H&E staining of a lung cancer metastasis to the brain. (B) Clusters of gene expression within the tissue. (C) scRNA-seq was performed on CD4^+^ T cells from three patients. Cells are plotted based on their UMAP dimensionality reduction coordinates and colored by gene expression cluster. (D) Expression of selected genes. (E and F) Spatial localization of a granzyme-expressing CD4^+^ T cell clone in (E) and multiple Treg clones in (F). (G and H) scRNA-seq phenotype of these clones are shown in panels (G and H).

An example implementation of these data is shown in Figure 4. A lung cancer metastasis to the brain - patient 27 from (Sudmeier et al., 2022) - was stained with hematoxylin and eosin (Figure 4A) and subjected to the standard Visium gene expression assay and spatial TCR method as described above. Gene expression analysis revealed seven clusters of spatial gene expression (Figure 4B). scRNA-seq on FACS-sorted CD4^+^ T cells was performed in parallel, identifying seven clusters of CD4^+^ T cells, including regulatory T cells (Tregs), granzyme-expressing cells, and naïve cells from a healthy donor (Figures 4C and 4D). TCR overlap was minimal between these scRNA-seq clusters (Figures 4G and 4H). CD4^+^ T cell clones identified by scRNA-seq were detectable in the spatial TCR results, including single and multiple clones associated with the Treg and granzyme-expressing phenotypes (Figures 4E and 4F). In (Sudmeier et al., 2022), we use this technique to show that TCR clonotypes expressed by exhausted CD8^+^ T cells localize to the tumor parenchyma.

## Limitations

The quality of spatial TCR libraries resulting from this protocol will depend primarily on two critical factors. First, the quality of cDNA from the Visium gene expression protocol used as input into this method will affect TCR sequence recovery. For example, low diversity libraries resulting from under-permeabilized tissue will negatively impact TCR sequences recovered by this method. Over-permeabilized tissue may result in diffusion of cDNA and less precise spatial localization. Second, tissues that are poorly infiltrated by T cells will not yield TCR cDNA, even when input cDNA is of high quality.

## Troubleshooting

### Problem 1

No amplification of TCR sequences.

If no PCR product is obtained after step 19, this may indicate poor quality of input Visium gene expression cDNA or a lack of T cell infiltration.

### Potential solution

• Check quality of Visium gene expression cDNA with a Bioanalyzer or similar electrophoresis technique. If quality is poor, optimization of the Visium gene expression protocol is required.
• Assess T cell infiltration in the tissue section. Tissue sections that are not infiltrated by T cells will not yield TCR cDNA. T cell infiltrate can be directly assessed by immunofluorescence or immunohistochemical staining of an adjacent tissue slice with an anti-CD3 antibody. Alternatively, Visium gene expression of the *CD3E* gene (or *CD8A* or *CD4* genes for CD8^+^ and CD4^+^ T cells, respectively) can be used as a proxy for T cell infiltration within the assayed tissue (see Figure 3A).

### Problem 2

Poor sequencing quality.

Poor sequencing quality in step 24 may be due to challenges presented by the low diversity of the spatial libraries generated by this protocol.

### Potential solution

• Ensure appropriate spike-in of PhiX Control library (Illumina). PhiX has a diverse base composition, helping to balance fluorescence signals during sequencing of low diversity libraries. We used 10% during MiSeq sequencing, but this may need to be increased for other Illumina sequencing instruments or for particularly low diversity libraries (such as tissues with extremely low TCR abundance or diversity).
• MiSeq and HiSeq Illumina sequencing instruments use 4-channel chemistry that may be better equipped to sequence low diversity libraries. For instruments with 2-channel chemistry, such as MiniSeq or NextSeq platforms, a higher amount of PhiX spike-in may be needed (Illumina, 2021).

### Problem 3

No detection of TCR sequences by MiXCR.

If few TCRs are detected by MiXCR in steps 25 and 26, this may indicate poor quality of input Visium gene expression cDNA, a lack of T cell infiltration, or inappropriate parameter inputs to the MiXCR software.

### Potential solution

• Similar to the solutions for Problem 1 above, check quality of Visium gene expression cDNA and assess T cell infiltration in the tissue section.
• Paired-end sequencing of this protocol results in two FASTQ files: read 1, which contains spatial barcode and UMI information, and read 2, which contains TCR sequences. Ensure that the read 2 FASTQ file has been input to MiXCR.
• Ensure that the correct species has and input material have been input to the MiXCR pipeline; use “-s hsa” for human sequences and “--starting-material rna” for Visium cDNA libraries.

### Problem 4

Identification of B cell receptor clones by MiXCR.

For some samples, we observed a large number of B cell receptor (BCR) clones called by MiXCR, especially light chain genes (*IGKL* and especially *IGKV*) in steps 25 and 26. Depending on the tissue used in this protocol, large amounts of BCR mRNA may be present in the original sample, depending on the B cell types present. Recent studies have identified BCR sequences in lymphoid tissues using the Visium platform using a deep sequencing approach (as opposed to targeted amplification) (Meylan et al., 2022).

We believe identification of BCR sequences with our protocol is due to homology between V gene segments and represents actual BCR sequences found in tissue. However, it is unlikely that these sequences are representative of the actual BCR repertoire in the tissue.

### Potential solution

• Ensure that only TCRβ genes are used in downstream analysis after step 26.

### Problem 5

TCRs detected outside of tissue-covered slide regions.

After completion of step 28, if TCR sequences have been detected in Visium capture spots that are not covered by tissue, this likely indicates problems with permeabilization of the tissue during Visium gene expression cDNA generation. Alternatively, an incorrect tissue positions list from Space Ranger output may have been used to map TCR sequences to capture spots.

### Potential solution

• Assess the “Fraction Reads in Spots Under Tissue” in the Web Summary output by Space Ranger during analysis of the Visium Spatial Gene Expression sequencing. If this number is low, this may indicate large amounts of ambient RNA or an inappropriate permeabilization time during Visium gene expression cDNA generation.
• Ensure that the spatial positions list used during analysis in steps 27–28 is matched to the sample output by Space Ranger. This file is typically found at “spatial/tissue_positions_list.csv” in Space Ranger output.

## Resource availability

### Lead contact

Further information and requests for resources and reagents should be directed to and will be fulfilled by the lead contact, William Hudson (william.hudson@emory.edu).

### Materials availability

This study did not generate new unique reagents.

## Data Availability

• Single-cell RNA-seq data and spatial transcriptomics have been deposited at GEO and are publicly available as of the date of publication. Spatial TCR-seq reads have been deposited in the SRA. Microscopy images from spatial transcriptomics are publicly available in the GEO deposition. Accession numbers and DOIs are listed in the key resources table. • Code used to identify and map TCR sequences from spatial transcriptomics data has been deposited on GitHub. The accession numbers and DOIs are listed in the key resources table. • Any additional information required to reanalyze the data reported in this paper is available from the lead contact upon request.

## Supplemental information

Table S1. Primers used for TCR amplification, related to “before you begin”, step 2

## References

[bib1] Bolotin D.A., Poslavsky S., Mitrophanov I., Shugay M., Mamedov I.Z., Putintseva E.V., Chudakov D.M. (2015). MiXCR: software for comprehensive adaptive immunity profiling. Nat. Methods.

[bib2] Dash P., McClaren J.L., Oguin T.H., Rothwell W., Todd B., Morris M.Y., Becksfort J., Reynolds C., Brown S.A., Doherty P.C., Thomas P.G. (2011). Paired analysis of TCRα and TCRβ chains at the single-cell level in mice. J. Clin. Invest..

[bib3] 10x Genomics (2022). Visium spatial gene expression reagent kits user guide. https://support.10xgenomics.com/permalink/tU6hscmgBOkYrT8OorjWw.

[bib4] Illumina (2021). What is nucleotide diversity and why is it important?. https://support.illumina.com/bulletins/2016/07/what-is-nucleotide-diversity-and-why-is-it-important.html.

[bib5] Meylan M., Petitprez F., Becht E., Bougoüin A., Pupier G., Calvez A., Giglioli I., Verkarre V., Lacroix G., Verneau J. (2022). Tertiary lymphoid structures generate and propagate anti-tumor antibody-producing plasma cells in renal cell cancer. Immunity.

[bib6] Robins H.S., Campregher P.V., Srivastava S.K., Wacher A., Turtle C.J., Kahsai O., Riddell S.R., Warren E.H., Carlson C.S. (2009). Comprehensive assessment of T-cell receptor β-chain diversity in αβ T cells. Blood.

[bib7] Saligrama N., Zhao F., Sikora M.J., Serratelli W.S., Fernandes R.A., Louis D.M., Yao W., Ji X., Idoyaga J., Mahajan V.B. (2019). Opposing T cell responses in experimental autoimmune encephalomyelitis. Nature.

[bib8] Sudmeier L.J., Hoang K.B., Nduom E.K., Wieland A., Neill S.G., Schniederjan M.J., Ramalingam S.S., Olson J.J., Ahmed R., Hudson W.H. (2022). Distinct phenotypic states and spatial distribution of CD8^+^ T cell clonotypes in human brain metastases. Cell Rep. Med..

[bib9] Tiller T., Meffre E., Yurasov S., Tsuiji M., Nussenzweig M.C., Wardemann H. (2008). Efficient generation of monoclonal antibodies from single human B cells by single cell RT-PCR and expression vector cloning. J. Immunol. Methods.

[bib10] von Boehmer L., Liu C., Ackerman S., Gitlin A.D., Wang Q., Gazumyan A., Nussenzweig M.C. (2016). Sequencing and cloning of antigen-specific antibodies from mouse memory B cells. Nat. Protoc..

